# Bone-Marrow-Derived Mesenchymal Stem Cells for Organ Repair

**DOI:** 10.1155/2013/132642

**Published:** 2013-03-11

**Authors:** Ming Li, Susumu Ikehara

**Affiliations:** Department of Stem Cell Disorders, Kansai Medical University, Moriguchi, Osaka 570-8506, Japan

## Abstract

Mesenchymal stem cells (MSCs) are prototypical adult stem cells with the capacity for self-renewal and differentiation with a broad tissue distribution. MSCs not only differentiate into types of cells of mesodermal lineage but also into endodermal and ectodermal lineages such as bone, fat, cartilage and cardiomyocytes, endothelial cells, lung epithelial cells, hepatocytes, neurons, and pancreatic islets. MSCs have been identified as an adherent, fibroblast-like population and can be isolated from different adult tissues, including bone marrow (BM), umbilical cord, skeletal muscle, and adipose tissue. MSCs secrete factors, including IL-6, M-CSF, IL-10, HGF, and PGE2, that promote tissue repair, stimulate proliferation and differentiation of endogenous tissue progenitors, and decrease inflammatory and immune reactions. In this paper, we focus on the role of BM-derived MSCs in organ repair.

## 1. Introduction

The shortage of donor organs and the need of lifelong immunosuppression for the thousands of patients suffering from end-stage diseases worldwide are problems that need to be resolved. The repair, replacement, and regeneration of organs can restore impaired functions and are regarded as a potential solution to allotransplantation [1]. The bone marrow (BM) is an invaluable source of adult pluripotent stem cells, including hematopoietic stem cells (HSCs), endothelial progenitor cells (EPCs), and mesenchymal stem cells (MSCs). MSCs are prototypical adult stem cells with the capacity for self-renewal and differentiation with a broad tissue distribution. MSCs have been identified as an adherent, fibroblast-like population, originally isolated from BM [2]. These multipotent cells can be differentiated *in vitro* and *in vivo* into various cell types of mesenchymal origin, such as osteoblasts, adipocytes, and chondrocytes [3, 4]. Recently, more reports have demonstrated that MSCs secrete a variety of factors that promote tissue repair, stimulate proliferation and differentiation of endogenous tissue progenitors, and decrease inflammatory and immune reactions [5–7]. Because MSCs do not evoke an immune response, they are useful for allogenic organ and tissue repair.

## 2. Source, Multilineage Potential and Definition of MSCs

MSCs were first isolated from BM and have since been isolated from different adult tissues, including skeletal muscle [8], adipose tissue [9], umbilical cord [10], synovium [11], the circulatory system [12], dental pulp [13], amniotic fluid [14], fetal blood [15], lung [16], liver, and BM [17]. Friedenstein and coworkers first reported the existence of adherent, fibroblast-like cells isolated from BM [2], and that these cells could differentiate into mesodermal lineage such as osteoblasts, adipocytes, and chondrocytes *in vitro* [18] and cardiomyocytes [19]. Also, MSCs have been reported to differentiate into types of cells of endodermal and ectodermal lineages, including lung [20], retinal pigment [21], skin [22], sebaceous duct cells [23], renal tubular cells [24], and neural cells [25, 26], hepatocytes [27], and pancreatic islets [28]. There has hitherto been no specific surface marker for the identification of MSCs. For the isolation of human MSCs, the International Society for Cell Therapy proposed criteria [18] that comprise (1) adherence to plastic in standard culture conditions; (2) expression of the surface molecules CD73, CD90, and CD105 in the absence of CD34, CD45, HLA-DR, CD14 or CD11b, CD79a, or CD19 surface molecules as assessed by fluorescence-activated cell sorter analysis; (3) a capacity for differentiation to osteoblasts, adipocytes, and chondroblasts *in vitro*. Similarly, murine MSCs have been shown to differ from human MSCs in terms of marker expression and behavior and have been identified as an adherent, fibroblast-like population, negative for CD45, CD11b, and CD 31, and positive for Scal1 and CD106 [29].

## 3. MSCs and the Immune System

MSCs have the ability to modify and influence almost all the cells of the innate and adaptive immune systems, to interfere with and affect cellular proliferation, differentiation, maturation, and function to induce an anti-inflammatory phenotype, and to modulate the immune response mediated by MSC soluble factors, including IL-6, M-CSF, IL-10, TGF*β*, HGF, and PGE2 [7, 30, 31]. The innate immune cells include neutrophils, dendritic cells (DCs), natural killer (NK) cells, eosinophils, mast cells, and macrophages. MSCs modulate DC function, indirectly regulate T and B cell activities, delay and prevent the development of acute graft versus host disease (GVHD) [32], and suppress DC function during allogeneic islet transplantation [33]. MSCs have been shown to suppress these inflammatory cells [34] and to alter NK cell phenotype and suppress proliferation, cytokine secretion, and cytotoxicity against HLA class I expressing targets [35]. MSCs mediated NK cell suppression via soluble factors such as indoleamine 2,3-dioxygenase, PGE2, and TGF*β* [36]. The adaptive immune system, which is composed of T and B lymphocytes generates specific immune responses to pathogens with the production of memory cells. It has been reported that MSCs upregulate anti-inflammatory Th2 cytokines, including IL-3, -5, -10, and -13, and downregulate proinflammatory Th1 cytokines, including IL-1*α* and *β*, IFN*γ*, and TNF*α* [37]. MSCs induced an alteration of DC cytokine secretion, inducing a decreased secretion of pro-inflammatory cytokines such as TNF*α*, IFN*γ*, and IL-12, and increased IL-10, which is a suppressive cytokine and inducer of reg T cells [38]. MSCs exert an inhibitory effect on B cells, but MSCs have stimulatory effect in low doses [39]. Concerning the immunomodulatory properties of MSCs in a mouse model, one report [40] has suggested that allogeneic MSCs are not intrinsically immunoprivileged, and under appropriate conditions, allogeneic MSCs induce a memory T-cell response resulting in rejection of an allogeneic stem cell graft. Another report [41] has suggested that MSCs could potentially improve experimental autoimmune encephalomyelitis in mice.

## 4. Homing of MSCs

Intravenously injected MSCs can migrate to the BM [42, 43] in the steady state and home to the inflammation site by migrating across the endothelium and then entering the injured organ [20, 44–47]. The fact that MSCs confer protection cannot be entirely attributed to their ability to home and engraft to the site of damage, suggesting that they are also capable of mediating protection in an endocrine manner [1]. MSCs have many chemokine receptors that assist in their migration to inflammatory sites via the SDF1/CXCR4 pathway [48]. Moreover, studies have demonstrated that platelet-derived growth factor-AB, IGF-1, and CD44 are the most potent chemoattractants for MSCs [44, 49].

## 5. BM-Derived MSCs (BMMSCs) and Organ Repair

Many reports have indicated that MSCs have the capacity to differentiate into endodermal, mesodermal, and ectodermal lineage cells. Recently, a report has indicated that the ability of MSCs to alter the tissue microenvironment via the secretion of soluble factors may contribute more significantly than their capacity for differentiation in tissue repair [50]. Adipose tissue and BM are the most readily available sources of MSCs because they are easy to harvest, and because of their relative abundance of progenitors and the lack of ethical concerns. Although adipose tissue-derived MSCs and BMMSCs show the same immunoregulatory and supporting hematopoiesis [51], BMMSCs have a higher degree of commitment to differentiate into chondrogenic and osteogenic lineages than adipose tissue-derived MSCs [52]. BMMSCs have been shown to ameliorate tissue damage and to improve function after lung injury [53–55], kidney disease [56, 57], diabetes [58, 59], myocardial infarction [60, 61], liver injury [62, 63], and neurological disorders [64]. 

### 5.1. BMMSCs and Lung

The lung is an organ that is highly susceptible to edema and endothelial permeability after traumatic injury. BMMSCs inhibit endothelial cell barrier permeability and preserve pulmonary endothelial cell integrity by preserving adherent junctions, tight junctions and decreasing inflammation. BMMSCs address both components of endothelial permeability and inflammation induced by hemorrhagic shock [54]. Interstitial lung diseases are characterized by epithelial injury, fibroblast proliferation, expansion of the lung matrix, and dyspnea. Of these diseases, idiopathic pulmonary fibrosis (IPF) is the most frequent and lethal. Proinflammatory cytokines IL-1 and TNF-*α* induce endothelial cells to express adhesion molecules and chemokines that attract other white cells from the blood to the site of injury [65]. IL-1 and TNF-*α* also stimulate proliferation of endothelial cells and fibroblasts that increase the blood supply at the site of injury and repair damage by the formation of scar tissue [66]. BMMSCs protect lung tissue from bleomycin-induced injury by blocking TNF-*α* and IL-1, two fundamental proinflammatory cytokines in the lung [53]. BMMSCs enhance the restoration of systemic oxygenation and lung compliance and decrease lung inflammation and histological lung injury. They also secrete cytokines, enhance lung repair, and attenuate the inflammatory response following ventilator-induced lung injury [55].

### 5.2. BMMSCs and Kidney

Acute and chronic kidney injuries after transplantation have a complex pathophysiology involving ischemic, inflammatory, and immunologic mechanisms, and adult stem cells have been used in the treatment of these kidney diseases. Adult BM stem cells and the kidney precursors have been demonstrated to have an ability to differentiate into the kidney's specialized structures [67]. Nephrons are of mesenchymal origin, and stromal cells are of crucial importance for signaling, leading to the differentiation of both nephrons and collecting ducts [67]. Ischemic acute renal failure (ARF), characterized by a sharp decline in the glomerular filtration rate, is a very common complication in hospitalized patients and particularly in patients with multiorgan failure. When BMMSCs are injected after ARF, they can histologically become located in the kidney and significantly enhance the recovery of renal function by transdifferentiation into renal tubular or vascular endothelial cells [24, 68]. A single intrarenal administration of BMMSCs 7 days after ischemia-reperfusion significantly improved renal function and modified renal remodeling. The improvement of renal function was associated with a reduction in extracellular matrix accumulation. In addition, MSC administration also reduced tubular dilation, which is a classical feature of progressive renal failure in a renal ischemia rat model [57]. 

### 5.3. BMMSCs and Pancreas

Diabetes is caused by absolute insulin deficiency due to autoimmune destruction of insulin-secreting pancreatic *β*-cells (type 1 diabetes) or by relative insulin deficiency due to decreased insulin sensitivity, usually observed in overweight individuals (type 2 diabetes). In both types of the disease, an inadequate mass of functional *β*-cells is the major determinant for the onset of hyperglycemia and the development of overt disease. BM and BMMSCs induce the regeneration of recipient-derived pancreatic insulin-secreting cells, and MSCs inhibit T-cell-mediated immune responses against newly formed *β*-cells, which are able to survive in this altered immunological milieu [69]. 

Acute pancreatitis (AP) is characterized by a rapid onset and disease progression, with high fatality. Pancreatic acinar cells are the functional unit for the external secretion of the pancreas, which accounts for 80% of pancreatic tissue. During the process of severe AP, inflammatory mediators, metabolic products of arachidonic acid, and oxygen-derived free radicals enhance vascular permeability and cause tissue thrombosis and hemorrhage, thereby inducing necrosis of the pancreas [70]. BMMSCs can effectively relieve injury to pancreatic acinar cells and small intestinal epithelium, promote the proliferation of enteric epithelium and repair of the mucosa, and attenuate systemic inflammation in rats with severe acute peritonitis [71]. 

Human BM stem cells are able to differentiate into insulin-expressing cells *in vitro* by a mechanism involving several transcription factors of the *β*-cell developmental pathway when cultured in an appropriate microenvironment [72]. Human BMMSCs can be induced to express insulin in sufficient quantities to to reduce blood glucose in a diabetic mouse model [73] and to protect human islets from proinflammatory cytokines [74]. The use of human BMMSCs could be developed as a cell therapy for pancreatitis because of the ability, as shown in a rat model of acute pancreatitis, to reduce inflammation and damage to pancreatic tissue by reducing levels of cytokines and inducing Foxp3(+) regulatory T cells [75]. 

### 5.4. BMMSCs and Heart

Cardiovascular diseases are the first cause of death worldwide, and myocardial infarction (MI) is responsible for 12.8% of all deaths [76]. BMMSCs have been shown to differentiate into myogenic phenotype [77] and show a potent antifibrotic action, as their conditioned medium decreases cardiac fibroblast proliferation and the expression of collagen types I and III [78, 79] and increases the secretion of antifibrotic molecules such as matrix metalloproteinases 2, 9, and 14 [80]. BMMSCs exhibit the ability to differentiate into cardiomyocytes, smooth muscle cells, and endothelium in a swine model of chronic ischemic cardiomyopathy [81]. They have been shown to prolong survival compared with controls when hearts of Wistar rats were transplanted to Fisher 344 rats with intravenous MSC infusion [82]. Intravenous fusion of MSCs is the easiest and most practical method for delivery, though the MSCs must travel through the pulmonary circulation, where entrapment of cells is a concern [83]. Intracoronary infusion of stem cells is delivered with a standard over-the-wire balloon angioplasty catheter placed into the target coronary artery [84]. Injected BMMSCs improve cardiac function and reduce scar size in acute MI [85, 86]. Early-phase clinical trial data demonstrate that MSC therapy for post-MI is safe and has favorable effects on cardiac structure and function [87, 88].

### 5.5. BMMSCs and Liver

FGF-4 is one of the most important members of the fibroblast growth factor family; it can initiate the proliferation of mesodermal and endodermal cells and improve the development of fetal liver [89]. HGF is essential for the development of several epithelial organs and has been one of the most well-characterized cytokines for the stimulation of DNA synthesis in primary hepatocyte cultures and for liver development [90]. Oncostatin M is a member of the interleukin-6 family produced by hematopoietic cells and induces the differentiation of fetal hepatic cells, conferring various metabolic activities of adult liver [91]. These three factors participate in different developmental stages of the liver. FGF4, HGF, and oncostatin M have been shown to be key cytokines for hepatic differentiation from mouse BMMSCs [92]. Transplantation of BMMSCs alleviates GalN-induced acute liver injury in rats and stimulates the recovery systems, as evidenced by an earlier surge of cellular proliferation and differentiation into functional hepatocytes. IL-6 exerts hepatoprotective and mitogenic effects by stimulating the induction of acute-phase proteins as well as by suppressing apoptosis. Transplantation of BMMSCs could ameliorate acute liver injury. It promotes cell proliferation and organ repair, and the activation of the IL-6/gp130-mediated STAT3 signaling pathway via soluble IL-6 receptor is crucial in hepatic differentiation of BMMSCs [93]. 

Liver fibrosis is the excessive accumulation of extracellular matrix proteins, including collagen, that occurs in most types of chronic liver disease. Advanced liver fibrosis results in cirrhosis, liver failure, and portal hypertension, and often requires liver transplantation [94]. Although liver transplantation is by far the most effective treatment for liver cirrhosis, extensive clinical application of the technique is limited by the lack of donor organ availability [95]. Cell-based hepatocyte transplantation, a potential interventional procedure, provides an effective strategy and holds great promise for the treatment of impaired livers. BMMSCs can protect against experimental liver fibrosis through promotion of IL-10 expression in CCl4- or dimethylnitrosamine-induced rats [63, 96]. 

### 5.6. BMMSCs and Brain

The development of effective treatments for human brain and spinal cord injury remains a serious challenge. In this regard, the transplantation of stem cells may help repair injured nerve tissue through the replacement of damaged cells, neuroprotection, or the creation of an environment conductive to regeneration by endogenous cells [97]. BMMSCs have been shown to promote cell proliferation and neurotrophic function of Schwann cells *in vitro* and *in vivo* [98]. Transplantation of BMMSCs can significantly reduce the behavioral abnormalities of these animals during the six weeks after engraftment [64]. Intravenously transplanted MSCs are capable of improving functional recovery and restoring neurological deficits in experimental intracerebral hemorrhage. The mechanisms are associated with enhanced survival and differentiation of neural cells and increased expression of antiapoptotic proteins and atrophic factors [99]. Human BMMSCs can improve neurological functional recovery in mice with experimental autoimmune encephalitis, possibly via a reduction of inflammatory infiltrates and areas of demyelination, stimulation of oligodendrogenesis, and by elevating brain-derived neurotrophic factor (BDNF) expression [41, 100]. Human BMMSCs transfected with the BDNF gene also showed improved functional recovery and reduced infarct size through a reduction in apoptosis [101]. Patients with Parkinson's disease transplanted with BMMSCs in the early stages of the disease (less than 5 years) showed greater improvement than in the later stages (11–15 years) [102].

### 5.7. BMMSCs and Intestine

Inflammatory bowel disease comprises a spectrum of chronic and relapsing diseases, including Crohn's disease (CD) and ulcerative colitis [103]. CD is characterized by a background of mucosal T-cell dysfunction, inflammatory cell infiltration, and abnormal cytokine production leading to uncontrolled and persistent intestinal transmural inflammation. Intraperitoneally injected cryopreserved BMMSCs home to and engraft into the inflamed colon and ameliorate trinitrobenzene sulfonic acid-induced colitis in rats [104]. Similarly, the injection of adipose-derived MSCs facilitated colonic mucosal repair and reduced the infiltration of inflammatory cells in the experimental colitis model [105].

Small intestinal permeability and villi injuries were significantly reduced in an MSC-administered group compared with the control group. MSC administration accelerated the recovery of the intestinal barrier dysfunction in a rat model of ischemia/reperfusion injury [106]. 

### 5.8. BMMSCs and Bone

Bone is regarded as an organ, and small bone damage can repair spontaneously without intervention. However, bone transplantation and surgery are required when there is extensive bone damage. As adult stem cells, BMMSCs possess a number of characteristics that make them appropriate for use in promoting bone regeneration [107]. BMMSCs may differentiate into tissue cells in order to restore lost morphology as well as function and to secrete a wide spectrum of bioactive factors that help to create a repair environment through their antiapoptotic effects, immunoregulatory function, and the stimulation of endothelial progenitor cell proliferation [108]. One report shows that BMMSCs stimulate growth with osteogenesis imperfecta when children received allogeneic BMMSCs [109].

## 6. Conclusion

**Figure 1:**
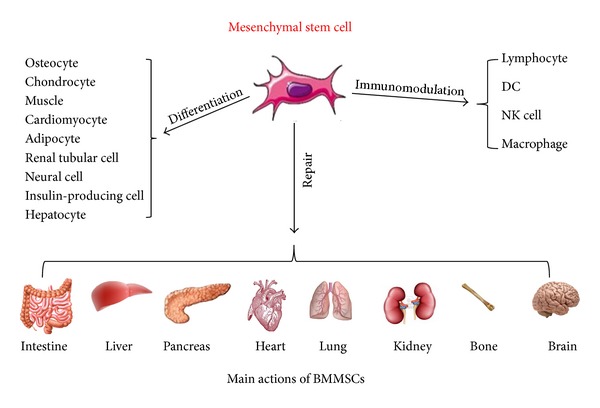
Main actions of BMMSCs.

Figure 1 summarizes the main actions of BMMSCs. The original use of BMMSCs was to accelerate hematopoiesis, since they have the potential to differentiate into various cells, and to secrete cytokines and growth factors. BMMSCs have immunomodulatory properties through paracrine and endocrine mechanisms to repair damaged tissue. Homing and immunomodulation are important aspects of MSC functioning and their clinical effects. It has been proposed that the anti-inflammatory and antiapoptotic effects of MSCs may promote tissue regeneration. The use of allogenic nonimmunogenic BMMSCs would be a more acceptable strategy clinically. The potential role of BMMSCs to promote engraftment of organs and prevent rejection may be multifactorial and might be dependent on secretion of soluble growth factors, increasing angiogenesis, suppressing alloreactive T cells, and interacting with several arms of the immune system. However, the long-term safety of transplanted BMMSCs for organ repair needs to be proven prior to their clinical application.
